# Ciliate and bacterial communities associated with White Syndrome and Brown Band Disease in reef-building corals

**DOI:** 10.1111/j.1462-2920.2012.02746.x

**Published:** 2012-08

**Authors:** Michael Sweet, John Bythell

**Affiliations:** School of Biology, Ridley Building, Newcastle UniversityNewcastle upon Tyne, NE1 7RU, UK

## Abstract

White Syndrome (WS) and Brown Band Disease (BrB) are important causes of reef coral mortality for which causal agents have not been definitively identified. Here we use culture-independent molecular techniques (DGGE and clone libraries) to characterize ciliate and bacterial communities in these diseases. Bacterial (16S rRNA gene) and ciliate (18S rRNA gene) communities were highly similar between the two diseases. Four bacterial and nine ciliate ribotypes were observed in both diseases, but absent in non-diseased specimens. Only one of the bacteria, *Arcobacter* sp. (JF831360) increased substantially in relative 16S rRNA gene abundance and was consistently represented in all diseased samples. Four of the eleven ciliate morphotypes detected contained coral algal symbionts, indicative of the ingestion of coral tissues. In both WS and BrB, there were two ciliate morphotypes consistently represented in all disease lesion samples. Morph1 (JN626268) was observed to burrow into and underneath the coral tissues at the lesion boundary. Morph2 (JN626269), previously identified in BrB, appears to play a secondary, less invasive role in pathogenesis, but has a higher population density in BrB, giving rise to the visible brown band. The strong similarity in bacterial and ciliate community composition of these diseases suggests that they are actually the same syndrome.

## Introduction

The emerging ‘damage-response’ framework of microbial pathogenesis (Casadevall and Pirofski, 2003) suggests that diseases in general arise from complex host–pathogen interactions. Lesser and colleagues (2007) argued that coral diseases in particular may result more commonly from environmentally induced changes in these host–pathogen interactions than the novel exposure of a host to a specific, virulent pathogen. Indeed, several proposed causal agents of coral disease, such as *Vibrio coralliilyticus* (Ben-Haim *et al*., 2003; Sussman *et al*., 2008), *V. shiloi* (Kushmaro *et al*., 2001) and *V. harveyi* (Luna *et al*., 2010), have commonly been detected in apparently healthy corals (Bourne, 2005; Bourne and Munn, 2005; Ritchie, 2006; Cervino *et al*., 2008; Kvennefors *et al*., 2010; Mouchka *et al*., 2010), increasing in abundance during disease and/or stress. In fact, it has been argued that all infectious agents could be considered ‘opportunistic’ and immunocompetent organisms may normally host many pathogens (defined as microorganisms *capable* of causing damage to the host; Casadevall and Pirofski, 2003). It is therefore vital that, in addition to the identification of pathogens via tests of Koch's postulates: (i) an analysis of the microbial community of healthy and diseased hosts is undertaken to comprehensively identify potential pathogens involved in disease, and (ii) increases in activity of these suspected pathogens are linked to sites of active pathogenesis. These need to be studied in combination to fully understand disease causation. Specifically we must be able to distinguish between pathogens that are capable of causing damage, those that are directly involved in a specific pathogenesis and heterotrophs that colonize dead and decaying tissues following disease.

Historically, most studies of coral diseases have been focused on pathogenic bacteria (Richardson *et al*., 1998; Kushmaro *et al*., 2001; Ben-Haim and Rosenberg, 2002; Patterson *et al*., 2002; Frias-Lopez *et al*., 2003; Cervino *et al*., 2008; Sussman *et al*., 2008; Luna *et al*., 2010). Only relatively recently have ciliates and other protozoans been shown to be associated with diseases of corals such as skeletal eroding band (SEB) (Antonius and Lipscomb, 2001) and Brown Band Disease (BrB) (Willis *et al*., 2004). BrB is widespread in parts of the GBR and known to affect at least three major coral families, including members of the *Acroporidae*, *Pocilloporidae* and *Faviidae* (Willis *et al*., 2004). A ciliate, identified as a member of the subclass *Scuticociliatia* (Bourne *et al*., 2008), has been shown to ingest intact symbiotic algae of the coral and isresponsible for the visible signs of this disease (a variable brown band). In 2006, ciliates (*Halofolliculina* sp.) were also reported affecting over 26 Caribbean reef-building coral species (Croquer *et al*., 2006a). Although it is still to be determined whether this Caribbean Ciliate Infection (CCI) is the same as SEB in the Indo-Pacific, their morphology, life cycle and patterns of infection are similar. Therefore, increasing evidence indicates that ciliates act as pathogens in some coral diseases. Despite this, Koch's postulates have not been fulfilled for any of the ciliates associated with coral diseases. However, several studies have shown ciliates to be pathogenic in a wide range of other organisms (Song and Wang, 1993; Bradbury, 1996), including members of the *Scuticociliatia* affecting marine mammals such as dolphins and whales (Sniezek *et al*., 1995; Poynton *et al*., 2001; Song *et al*., 2009) and members of the *Peritrichida* affecting bivalves such as the clam *Mesodesma mactroides* (Cremonte and Figueras, 2004).

Some of the most ecologically important coral diseases worldwide are the poorly defined ‘white diseases/syndromes’, few of which have been satisfactorily characterized (Bythell *et al*., 2004). These diseases are collectively termed White Syndrome (WS) in the Indo-Pacific and include White Plague (WP) and White Band Disease (WBD) in the Caribbean. Many studies have identified bacterial pathogens involved in these white diseases (Peters *et al*., 1983; Barash *et al*., 2005; Thompson *et al*., 2006; Sussman *et al*., 2008; Efrony *et al*., 2009). For example, *Aurantimonas coralicida* has been reported to cause WP type II disease (Denner *et al*., 2003) and another α-proteobacterium, thought to be the causative agent of juvenile oyster disease (JOD), has been associated with a WP-like disease (Pantos *et al*., 2003). Several vibrio species have been proposed as causal agents of WS (Sussman *et al*., 2008), with *V. harveyi* being the most recently identified (Luna *et al*., 2010). However, recently, Work and Aeby (2011) have reported that ciliates are also associated with the WS pathology. Together with Ainsworth and colleagues (2007) they also show no bacterial populations associated with the pathogenesis and no signs of bacterial-induced necrosis. These recent studies therefore question the primary role of bacteria in WS.

**Fig. 1:**
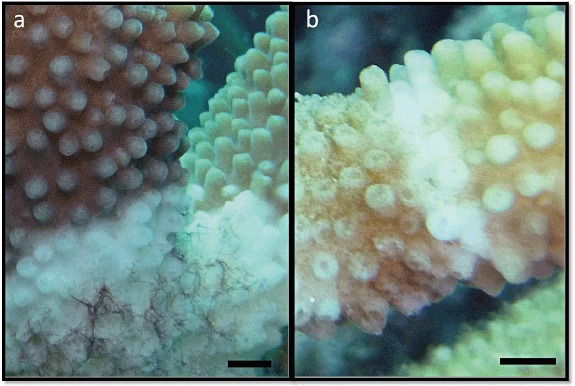
Photographs of *Acropora muricata* at Heron island on the Great Barrier Reef exhibiting disease signs of White Syndrome (A) and Brown Band Disease (B). Scale bar = 2 mm.

As a first step towards understanding disease causation in WS (Fig. 1A) this study provides a comprehensive, culture-independent molecular analysis of both ciliate and bacterial communities associated with the disease. As a comparison with a known ciliate-associated syndrome, we also sampled corals displaying characteristic signs of BrB (Fig. 1B). Since WS is a collective term that may encompass both active and recovering lesions (Work and Aeby, 2011), and is easily confused with non-infectious causes such as predation, we monitored disease lesion progression in the field and selected only cases that showed actively progressing disease lesions, referred to here as ‘Progressive White Syndrome (PWS)’ to distinguish it from these other WS states.

## Results

### Bacterial 16S rRNA gene diversity

**Fig. 2:**
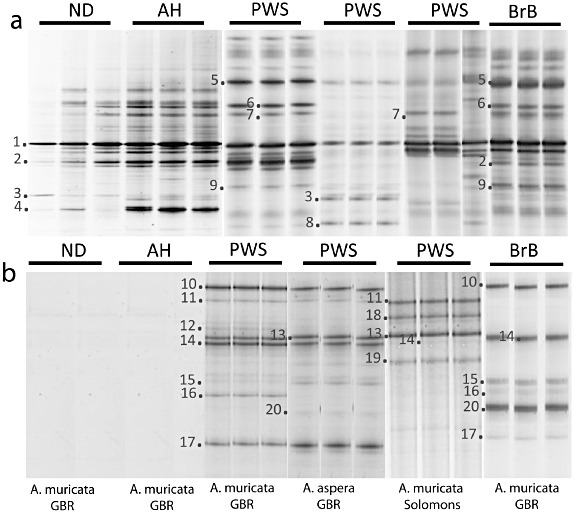
Representative denaturing gradient gel electrophoresis (DGGE) profiles of: ND: non-diseased coral; AH: apparently healthy tissue – the tissue above the advancing lesion on a disease coral; PWS: Progressive White Syndrome [note: from two different species, *Acropora muricata* and *A. aspera*, and from two different locations, Heron Island (GBR) and the Solomon Islands]; and BrB: Brown Band Disease; (A) bacterial 16S rRNA gene fingerprints (DGGE). Closest matches (GenBank accession numbers) from blast analysis: **1.** Symbiotic algal DNA, **2.***Endozoicomonas* sp. (DQ200474), **3.***Firmicutes* sp. (HQ444233), **4.***Aeromonas* sp. (HQ180147), **5.***Arcobacter* sp. (HQ317346), **6.***Vibrio harveyi* (GQ203118), **7.***Glycomyces* sp. (JF729475), **8.***Clostridium* sp. (GU227558), **9.***Cyanobacterium* sp. (FJ844162), and (B) ciliate 18S rRNA gene fingerprint; **10.***Diophrys* sp. (DQ35385), **11.***Pseudocarnopsis* sp. (HQ228545), **12.***Aspidisca* sp. (AF305625), **13.** Morph1 (FJ648350), **14.** Morph2 (AY876050), **15.***Euplotes* sp. (GU953668), **16.***Glauconema* sp. (GQ214552), **17.***Varistrombidium* sp. (DQ811090), **18.***Euplotes* sp. (AY361908), **19.***Hartmanula* sp. (AY378113), **20.***Holosticha* sp. (DQ059583). Composite DGGE image standardized for gel-to-gel comparison using BioNumerics.

Significant differences in denaturing gradient gel electrophoresis (DGGE) banding patterns of bacterial 16S rRNA gene diversity were shown between non-diseased colonies (ND; *n* = 10), the apparently healthy tissues adjacent to the disease lesion (AH; *n* = 10) and the disease lesion (DL; *n* = 10) in *Acropora muricata* from Heron Island, GBR [one-way analysis of similarity (ANOSIM), *R* = 0.937, *P* < 0.001]. There was no significant difference in bacterial 16S rRNA DGGE profiles between corals with PWS and those with BrB (ANOSIM, pairwise comparison, *P* = 0.64) of the same species from the same location; *n* = 10 and 12 respectively. Only four bacterial ribotypes were detected in diseased or apparently healthy tissue (tissue near the disease lesion) yet absent in non-diseased samples, including ribotypes related to *Clostridium* sp. (GenBank Accession No. JN406280), *Aeromonas* sp. (JN406279), *Cyanobacterium* sp. (JN406285) and *Arcobacter* sp. (JF831360). All four of these sequences were dominant representatives of both DGGE profiles (Fig. 2A) and clone libraries (Table 1), which were based on independent primer sets targeting different subregions of the 16S rRNA gene. One of these, *Arcobacter* sp. (JF831360), was absent in non-diseased tissues, appeared in apparently healthy tissues and increased substantially in relative 16S rRNA gene abundance in the disease lesion (Table 1). This species was also consistently represented in all replicate samples of the disease (Fig. 2A). The other three ribotypes did not increase as markedly in relative abundance (Table 1) and were not consistently the dominant ribotypes across replicate samples (Fig. 2A). Ribotypes related to *Glycomyces* sp. (JN406287), *V. harveyi* (JN406288), *Microbacterium* sp. (JN406289), *Ferrimonas* sp. (JN406292), *Cyanobacterium* sp. (JN406296), *Pseudoalteromonas* sp. (JN406297), *Shewanella* sp. (JN406298) and a *Marinobacter* sp. (JN406299) were all present in clone libraries of non-diseased corals in low relative abundance (Table 1), but increased both in apparently healthy tissue and at the disease lesion itself. Interestingly, one ribotype related to *Aeromonas* sp. (JN406293) increased in dominance in AH but decreased again in all disease lesion samples (Fig. 2A; Table 1). Four out of these nine ribotypes (*Glycomyces* sp., *V. harveyi*, *Cyanobacterium* sp. and the *Aeromonas* sp.) were also detected as dominant DGGE bands in the apparently healthy or diseased samples.

**Table 1:** Heatmap summarizing the relative abundance (%) of dominant bacterial sequence affiliations for 16S rRNA gene clone libraries

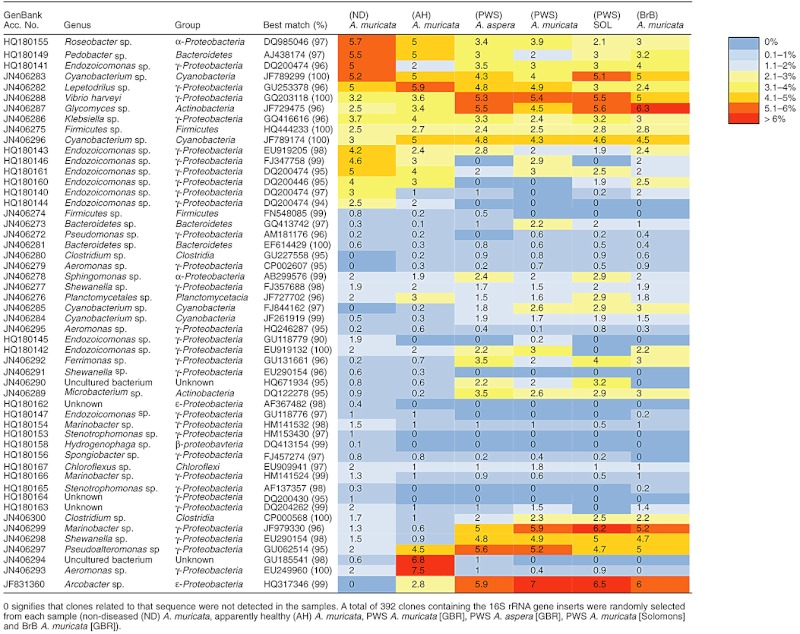

### Microscopic and molecular identification of ciliates

**Fig. 3:**
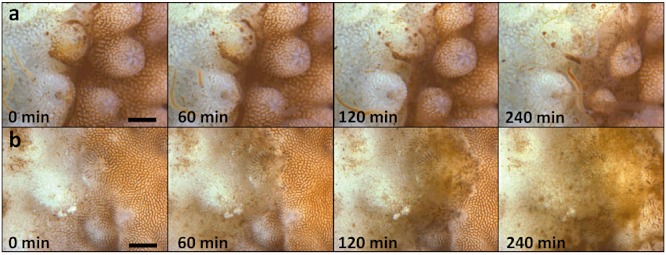
Time-lapse images of PWS (A) and BrB (B) lesion progression. The lesion progresses from left to right of the images. At this scale, individual ciliates are difficult to distinguish. (A) The ciliate mass appears to be a diffuse yellow-brown mass comprised predominantly of the rapidly moving Morph1 (JN626268) ciliates embedded with variable densities of Morph2 (JN626269) ciliate, while the BrB lesion (B) is dominated by the ciliate Morph2 (JN626269). These are slower moving and large enough to be seen as individual cells, typically orientated perpendicularly to the coral skeleton surface (white) exposed by the advancing lesion. Coral tissues (yellow-brown) immediately adjacent to the advancing lesion are intact and appear normally pigmented. Scale bar = 1 mm.

Live microscopic examination of all PWS and BrB lesions over time (Fig. 3), showed diverse communities of ciliates, in large, mobile population masses at the edge of the disease lesions, adjacent to apparently healthy tissues and recently exposed coral skeleton (Table 2; Figs 2B and 3). Pathogenesis was observed via time-lapse videography (Videos S1 and S2) and a number of ciliate morphotypes were observed to actively engulf coral tissues at the site of lesion progression. Ciliate communities associated with progressive disease lesions encompassed at least 11 different ciliate morphotypes (Table 2), four of which contained coral endosymbiotic algae, indicative of coral tissue ingestion. There were no ciliates observed or detected by molecular screening in samples of non-diseased and apparently healthy coral samples (Fig. 2B). There was no significant difference (ANOSIM, *R* = 1, *P* = 0.12) between DGGE profiles of ciliate 18S rRNA gene diversity between coral species (*A. muricata*, *n* = 10; and *A. aspera*, *n* = 4) for PWS. However there was a significant difference (ANOSIM, *R* = 0.56, *P* = 0.04) between *A. muricata* with PWS collected from the GBR (*n* = 10) and those from the Solomon Islands (*n* = 5) (Fig. 2B). This difference was due to the lack of some species, such as *Euplotes* sp. (JN406271), *Glauconema* sp. (JN406267), *Holosticha* sp. (HQ013356), *Varistrombidium* sp. (HQ204551) and *Diophrys* sp. (JN406270)in the Solomon Islands samples compared with those from the GBR (Fig. 2B). Significant differences (ANOSIM, *R* = 1, *P* = 0.04) also occurred between PWS and BrB samples from the same reef site and same coral species, *A. muricata*. However, the two dominant ciliates, Morph1 (JN626268) and Morph2 (JN626269), were present in both BrB and PWS disease lesions and at both locations (GBR and Solomon Islands), but at different population densities.

**Table 2:** Morphological descriptions of the ciliates visually observed to be associated with PWS and BrB diseased corals, showing the species ID from single cell isolates, closest match and GenBank accession number, a unique GenBank accession number for each ciliate sequence from this study and a photograph of each ciliate described

Species ID based on morphology	Accession No.	Closest match (%)	Description	Photo ID	PWS	BrB
*Morph1;*	HQ204545/JN626268	FJ648350 (99)	Body slender, 60–200 × 20–60 µm *in vivo*, variable in outline from cylindrical to fusiform; anteriorly narrowed and conspicuously pointed. Length of buccal field ∼ 40–50% of body, cytostome conspicuous and deeply sunk. Pellicle rigid, packed with close-set extrusomes (*c*. 2–3 µm long). Macronucleus band-like, twisted and positioned centrally along cell median with several micronuclei attached to it. One small, terminally located contractile vacuole. Approximately 50 somatic kineties composed of monokinetids, with cilia *c*. 7–10 µm long; oral cilia ∼ 10–15 µm long; caudal cilium 12–15 µm in length. Paroral membrane L-shaped, on right of oral cavity, slightly oblique to main body axis. Scutica with *c*. 15 basal bodies. Extrusomes densely packed beneath pellicle. Locomotion by fast, spiral swimming while rotating irregularly about its main body axis, motionless for short periods when feeding. Slight (up to 0.5%) genetic variation in DNA sequence between individuals sampled.	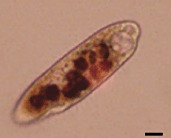	✓	✓
*Philaster* sp.						
*Morph2;*	HQ204546/JN626269	AY876050 (100)	Body larger, 200–500 × 20–75 µm, variable in outline, cylindrical to fusiform; anteriorly rounded or slightly tapered. Oral depression was conspicuous and deeply invaginated, with right-posterior in-pocketing supported by fibres; the buccal field was ∼ 30–40% of cell length. The cytostome was clearly delineated by fibres, leading to a cytopharynx extending ∼ 30% of the cell length. The macronucleus was sausage-like, elongate but often bent, positioned centrally along the main cell axis. Micronuclei were not observed, as prey (coral zooxanthellae and nematocysts) nuclei obfuscated identification. Somatic cilia were ∼ 5 µm long; oral cilia ∼ 5–10 µm long, forming conspicuous polykinetids. Cells were colourless to brownish yellow, often with numerous food vacuoles or zooxanthellae. Division was rarely noted in preserved specimens but commonly seen. Slight (up to 0.5%) genetic variation in DNA sequence between individuals sampled.	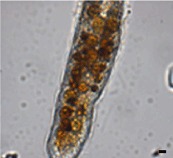	✓	✓
*Porpostoma guamense*						
*Aspidisca* sp.	JN406268	AF305625 (100)	Euplotine hypotrich ciliates, left-serial oral polykinetids separated during stomatogenesis; collar oral polykinetids in anterior ventral depression separated from lapel oral polykinetids in oral cavity; free-living, often sapropelic. No genetic variability between individuals sampled.	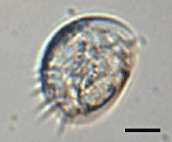	✓	
*Euplotes* sp.	JN406271	GU953668 (99)	Body slightly rectangular in outline, 90–140 µm long *in vivo*, with no conspicuous dorsal ridges. Length of buccal field about 65% of body. Cytoplasm hyaline, central area often dark due to food vacuoles and granules. Macronucleus C-shaped; micronucleus spherical. AZM with about 60 membranelles, proximal portion curved ∼ 90° to right. Ten frontoventral cirri in a genus-typical pattern; two left marginal cirri separated and aligned evenly with 3 or 4 caudal cirri. Nine or 10 dorsal kineties extending entire length of cell. Silverline system on dorsal side regular or irregular vannus-type. Locomotion by medium-fast crawling on coral, sometimes stationary for long periods. No genetic variability between individuals sampled.	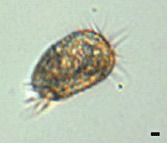	✓	✓
*Diophrys* sp.	JN406270	DQ353850 (99)	Body ∼ 70 × 25 µm *in vivo*, oval to slender oval with both anterior and posterior ends of body more or less pointed, grey to slightly yellowish. Ciliary organelles sometimes conspicuously long, especially the caudal cirri and the anterior adoral membranelles. Length of buccal field 40–50% of body. Ciliature typical of the scutum-mode: 5 frontal, 2 pretransverse ventral, 5 transverse and 3 caudal cirri. Two marginal cirri conspicuously separated from each other, posterior one always being below transverse cirri. Paroral membrane distinctly shorter than the endoral membrane. Three large genus-typical caudal cirri ∼ 4 dorsal kineties, the dikinetids of which are arranged in continuous rows. No genetic variability between individuals sampled.	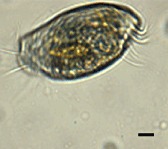	✓	✓
*Varistrombidium* sp.	HQ204551	DQ811090 (99)	Body 55–75 × 40–50 µm *in vivo*, slightly asymmetric and elongated barrel-shaped posterior end usually bluntly pointed; collar region domed to form a conspicuous apical protrusion; buccal cavity shallow and inconspicuous, extending obliquely to right and terminating ∼ 15–20% down cell; no hemitheca detected. Extrusomes prominent, acicular, *c*. 10 µm long, evenly arranged along dorsal side of cell and on narrowed upper equatorial and caudal areas, not in bundles. Macronucleus ovoid to ellipsoidal; micronucleus not found. AZM with distinct ventral opening and clearly divided into anterior and ventral parts comprising 15–17 and 7 or 8 membranelles respectively. Five somatic kineties (SK) composed of dikinetids. Slight (up to 1%) genetic variation in DNA sequence between individuals sampled.	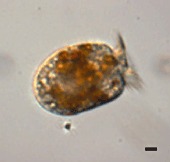	✓	✓
*Pseudokeronopsis* sp.	HQ013358	AY881633 (96)	Body 240–350 × 50–90 µm *in vivo*, dark reddish-colour, long elliptical with anterior end broadly rounded, posterior end narrowed, left margin conspicuously convex, right margin distinctly sigmoidal, widest in mid-region, dorsoventrally flattened;. Two types of cortical granule: type 1, pigmented orange, mainly grouped around cirri and dorsal bristles; type 2, colourless and blood-cell-shaped, lying just beneath type 1 granules and densely distributed. Approximately 9–13 pairs of frontal cirri in two rows forming a bicorona. Posterior end of bicorona continuous with long midventral row of 65–93 cirri that extends posteriorly to transverse cirri. Two fronto-terminal, one buccal and 7–11 transverse cirri, the latter forming a row that extends to anterior of cell; 48–79 left and right marginal cirri; 5–7 dorsal kineties. AZM with 68–92 membranelles and extends far onto right side of cell. One contractile vacuole positioned in posterior end of body. Numerous macronuclear nodules. Locomotion by slow crawling. Feeds on a variety of protists. Slight (up to 0.5%) genetic variation in DNA sequence between individuals sampled.	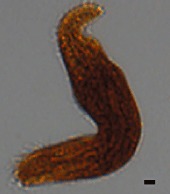	✓	✓
Holosticha sp.	HQ013356	DQ059583 (98)	Body 80–90 × 25–50 µm *in vivo*, generally fusiform with both ends slightly narrowed and dorsoventrally flattened. Cortical granules prominent, blood-cell-shaped and sparsely distributed. Three frontal, one buccal, two frontoterminal and 6–10 transverse cirri. Midventral row of 13–17 cirri extends to transverse cirri. One right and one left marginal cirral rows, the latter being obliquely bent at anterior end. Four dorsal kineties. Adoral zone with 24–30 membranelles including 8–13 distal membranelles that are separate from the others. Two ellipsoid macronuclear nodules. One contractile vacuole post-equatorially located and not easily observed. Locomotion mainly by crawling slowly on coral. Slight (up to 1%) genetic variation in DNA sequence between individuals sampled.	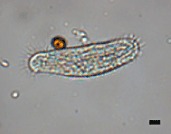	✓	✓
*Euplotes* sp.	HQ013357	AY361908 (95)	Body oval in outline, ∼ 60–70 × 50–60 µm *in vivo*, dorsoventrally flattened, with no conspicuous dorsal ridges. Length of buccal field ∼ 75% of body. Macronucleus C-shaped; micronucleus spherical. Proximal portion of AZM curved at ∼ 90° to right. Ten frontoventral, 5 transverse and 2 caudal cirri in genus-typical pattern; two left marginal cirri separated and aligned evenly with small caudal cirri. Eight to nine kineties extend entire length of cell, leftmost kinety containing only ∼ 5 dikinetids. Silverline system on dorsal side irregular vannus-type. Locomotion by slow, slightly jerky, crawling on coral, remaining stationary for long periods. No genetic variability between individuals sampled.	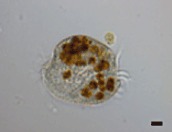	✓	✓
*Glauconema trihymene*	JN406267	GQ214552 (100)	Body 30–36 × 16–24 µm *in vivo*, bilaterally flattened with large apical plate in trophont, long oval to fusiform with small apical plate in tomite. Buccal cavity spacious in trophont while narrow in tomite. Pellicle thin, slightly notched. Single spherical macronucleus. Contractile vacuole positioned caudally. Seventeen somatic kineties, with cilia *c*. 8–10 µm long. Caudal cilium ∼ 15 µm long. Somatic kineties comprising mostly of dikinetids with only a few monokinetids in trophont; higher proportion of monokinetids in tomite. Locomotion by crawling slowly with frequent pauses in case of trophont or swimming quickly in case of tomite. No genetic variability between individuals sampled.	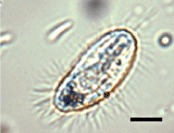	✓	✓
*Hartmannula derouxi*	JN406269	AY378113 (100)	Body 60–120 × 30–70 µm *in vivo*, long oval to elongate in outline. Pellicle covered with smooth, thin, colourless gel-like substance. Cyrtos with ∼ 30 nematodesmal rods. Many contractile vacuoles. Podite ∼ 20 µm long and secretes a glue-like substance for adhering to substratum. Forty-two to 53 somatic kineties, the rightmost 11–12 of which extend apically, comprising 12–19 right, 18–19 left and 10–15 suboral kineties. Approximately 13 kinetosome-like dots present near base of podite. Macronucleus ellipsoidal. Silverline system irregularly reticulate with several tiny argentophilic granules on or near silverlines.	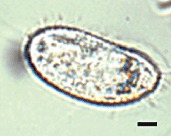	✓	

Scales bars on each photograph represent 10 µm.

The most aetiologically important agent in the mixed ciliate community in PWS samples appeared to be a ciliate closely related to a recently described member of a new genus, *Philaster* sp. (FJ648350) (Zhang *et al*., 2011). This ciliate (Morph1 JN626268) was approximately 60–80 µm long and 25–30 µm wide (Table 2). Movement in this morphotype was rapid and they were observed to actively burrow into and beneath the live coral tissues, which showed no signs of necrosis under the binocular microscope (Fig. 3A). This ciliate was seen in abundance at the lesion interface and was one of the four types containing coral algal symbionts (Table 2). In most cases, populations of this ciliate were mixed with populations of a larger (250–300 µm in length and 50 µm in width) ciliate Morph2 (JN626269), morphologically resembling a recently described ciliate, *Porpostoma guamensis* (Lobban *et al*., 2011), and identical in 18S rRNA gene sequence to the BrB ciliate (AY876050) identified by Bourne and colleagues (2008). This ciliate was also seen in abundance at the lesion interface and also contained algal endosymbionts from the coral (Table 2); however, it appeared to take a secondary role to Morph1 (JN626268). The movement of Morph2 (JN626269) was generally slower and less erratic than Morph1 (JN626268), with slow turning/spinning movements. A further two ciliate species, *Varistrombidium* sp. (HQ204551) and *Euplotes* sp. (HQ013357), dominated PWS samples (Table 2) and was seen to a lesser extent in BrB samples. These are relatively small species (55–70 µm in length), differing from the other morphotypes with prominent (∼ 10–12 µm) frontal cirri. The presence of symbiotic algae in these ciliate species again indicates ingestion of coral tissue. Other members of the mixed ciliate community present within PWS and BrB (Table 2) included a smaller ovoid ciliate, *Diophrys* sp. (JN406270), and the worm-like *Pseudokeronopsis* sp. (HQ013358); however, these were more commonly observed in the denuded coral skeleton rather than at the advancing tissue edge and none of these other ciliates contained coral symbionts or were observed ingesting coral tissues.

**Fig. 4:**
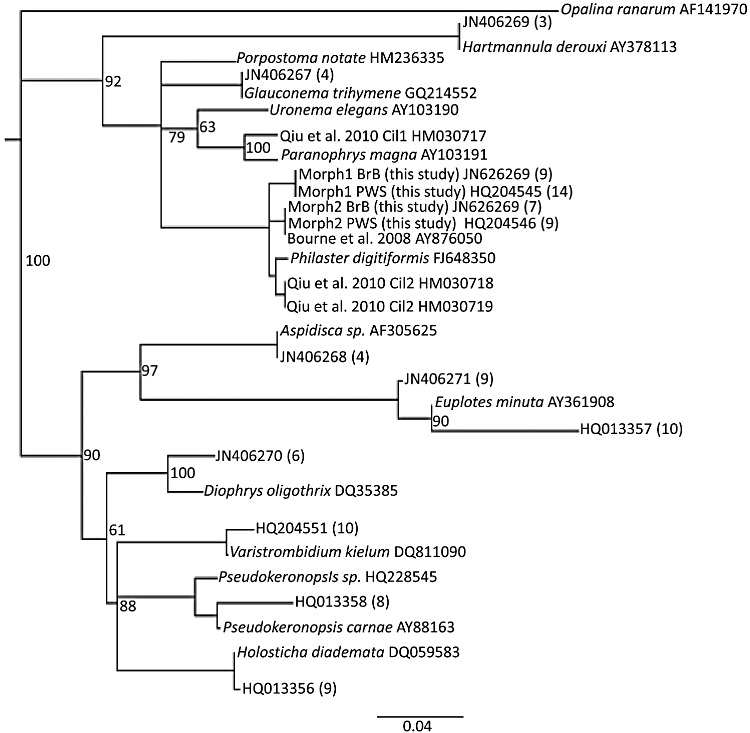
Neighbour-joining consensus tree of partial 18S rRNA gene sequences of 13 species of ciliates found within Brown Band Disease and Progressive White Syndrome. Number in brackets relates to number of sequences retrieved from single cell isolates. Sequences were aligned in clustal w2 (Larkin *et al*., 2007), using an IUB cost matrix with a gap open cost of 15 and a gap extend cost of 7. A neighbour-joining consensus tree (1000× re-sampling) was constructed in Geneious Pro 5.0 using the Tamura genetic distance model (Tamura, 1994) with an opalinid protist, *Opalina ranarium* (AF141970), as the outgroup.

**Fig. 5:**
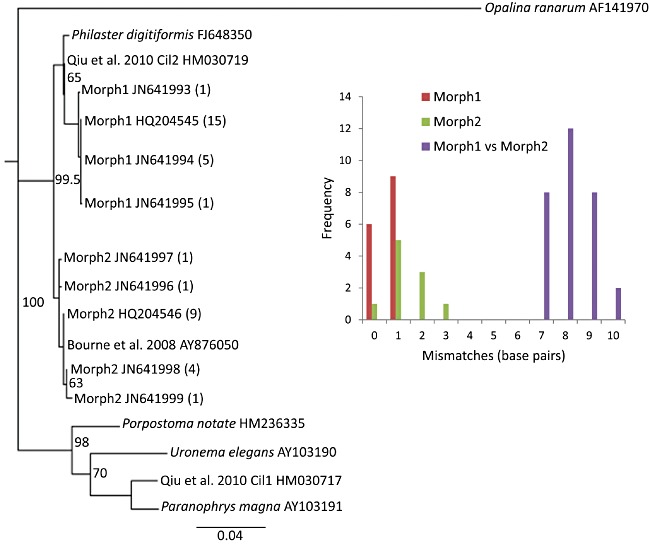
Neighbour-joining consensus tree of partial 18S rRNA gene sequences of 12 samples of Morph1 (JN626268) and Morph2 (JN626269) found within Brown Band Disease and White Syndrome, illustrating slight (up to 0.5%) variation within each morphotype, but 1.3–1.8% variation between morphotypes over 549 bp. Number in brackets relates to number of identical sequences obtained for the given GenBank accession number from single cell isolates of each morph. Sequence alignment and tree construction were as described in Fig. 4. Insert histogram shows sequence mismatch frequencies within and between sequences of Morph1 and 2.

18S rRNA gene sequences retrieved for Morph1 (JN626268) and Morph2 (JN626269) showed the two types to be closely related to each other and more closely related to *Philaster digitiformis* (FJ648350) than *Porpostoma notate* (HM236335) (Figs 4 and 5). Morph1 (JN626268) showed 99.3 ± 0.2% similarity to *P. digitiformis* (FJ648350) while Morph2 (JN626269) showed 98.7 ± 0.5% similarity (Fig. 5). Morph1 (JN626268) and Morph2 (JN626269) showed slight (up to 0.5%) variation in DNA sequence within each morph (Fig. 5). The two morphotypes shared 98.5 ± 0.1% similarity in a variable sequence region, with only 7–10 mismatches over 549 base pairs (Figs 5 and S1). However, there was strong bootstrap support (99.5%) in the neighbour-joining consensus tree for a phylogenetic separation between these morphotypes (Fig. 5). Nine out of sixteen sequences from Morph2 (JN626269) showed 100% sequence similarity to the ciliate identified by Bourne and colleagues (2008) from BrB disease, with the remaining seven differing by approximately 0.2%.

## Discussion

This article is the first to comprehensively describe the diverse ciliate communities associated with both WS and BrB and supports recent observations linking ciliates with PWS (Work and Aeby, 2011). Ciliates are important components of many microhabitats and are known to regulate microbial biomass (Vargas and Hattori, 1990) and bacterial community composition (Geltser, 1992), resulting in strong controls on both benthic and pelagic food webs (Fenchel, 1968; 1980; Porter *et al*., 1979; Wieltschnig *et al*., 2003; Vargas *et al*., 2007). This primary role of ciliates as bacterial feeders may have in the past led to their presence on corals being dismissed as secondary invaders (Ainsworth and Hoegh-Guldberg, 2009). Microscopic examination supports the conclusion that the ciliates associated with these diseases are largely responsible for the macroscopic signs of both PWS and BrB, namely an advancing lesion, with a sharp demarcation between visibly healthy tissues and the denuded skeleton (Bythell *et al*., 2004). The wide diversity of ciliate types in all the disease lesions sampled, including at least four that were observed to consume coral tissues, leads to effective clearance of tissues from the skeleton. However, we also observed at least one type (Morph1 JN626268) which appears to be involved in pathogenesis. This morphotype was consistently observed at the lesion interface and seen to burrow into and beneath apparently healthy tissues, as shown histologically by Work and Aeby (2011).

Surprisingly, the ciliate community associated with PWS was highly similar to that associated with BrB, except that the agent previously attributed to the visible signs of BrB by Bourne and colleagues (2008) (our Morph2 JN626269 = AY876050 of Bourne *et al*., 2008), was present in greater population densities within the brown band, a region that is often 1–2 mm away from the disease lesion boundary (Fig. 1B, Willis *et al*., 2004) and less dominant, but still consistently present in PWS samples. The characteristic brown band of BrB has been described as highly variable in ciliate population density and may not always be visible, leading to confusion with WS in field studies (Willis *et al*., 2004). In both PWS and BrB, the smaller, rapidly moving ciliate (Morph1) was more active at the disease lesion interface. Two other ciliates *Varistrombidium kielum* (HQ204551) and a *Euplotes* sp. (HQ013357) were also shown to contain coral endosymbionts, indicative of the ingestion of coral tissues, but these were not consistently observed in all cases of the diseases.

Morph1 (JN626268) was identified both morphologically and genetically as a member of the class *Scuticociliata*, closely related to *Philaster digitiformis* (FJ648350). Morph2 (JN626269) was morphologically similar to the ciliate associated with BrB described by Lobban and colleagues (2011) as *Porpostoma guamensis*. However, the 18S rRNA phylogeny shows this ciliate to be distinct from the only *Porpostoma* sp. sequenced to date, *Porpostoma notate* (HM236335), and more similar to Morph1 (JN626268) and *Philaster digitiformis* (FJ648350), and genetically identical to the BrB ciliate (AY876050) described by Bourne and colleagues (2008). It is therefore proposed that the proper epithet for this ciliate should be *Philaster guamensis* not *Porpostoma guamensis*. Morph2 (JN626269) also exhibited similar morphology and behaviour (orientation perpendicular to the coral skeleton surface), to that of the BrB ciliate described by Bourne and colleagues (2008).

Contrary to our expectations, given that WS is a broad descriptive term that has generally been assumed to encompass several distinct but visually similar diseases (Bythell *et al*., 2004; Sussman *et al*., 2008; Luna *et al*., 2010; Work and Aeby, 2011), the bacterial (16S rRNA gene) community structure associated with PWS was remarkably similar between independent replicate samples and also highly similar to the BrB bacterial (16S rRNA gene) community. In part, this was because we only sampled actively progressing lesions and so avoided including samples with arrested and recovering disease lesions (Work and Aeby, 2011) and/or possible non-pathogenic causes of mortality such as predation, which together comprised approximately 17% of apparent WS lesions observed in this study. This consistency between bacterial 16S rRNA gene diversity indicates that the progressive form of WS does not include a wide variety of disease types, at least at the locations and times studied.

One bacterial ribotype (*Arcobacter* sp. JF831360) increased consistently in all diseased samples but was absent in healthy coral. Similar *Arcobacter* sp. have previously been identified in Black Band Disease (Frias-Lopez *et al*., 2002; Sato *et al*., 2010) and WP (Sunagawa *et al*., 2009). Interestingly, this *Arcobacter* sp. (JF831360) appeared in apparently healthy tissues in advance of the disease lesion and so may be a candidate pathogen involved in active pathogenesis. Several other bacteria –*V. harveyi* (JN406288), *Glycomyces* sp. (JN406287), *Pseudoalteromonas* sp. (JN406297), *Shewanella* sp. (JN406298) and a *Marinobacter* sp. (JN406299) – were also identified in both PWS and BrB. These latter species increased in relative 16S rRNA gene abundance only at the disease lesion and not in advance of it, so although they are potential pathogens, they could equally be acting as heterotrophs colonizing dead and decaying tissues. Previously, several *Vibrio* species have been identified as potential WS pathogens (Sussman *et al*., 2008; Luna *et al*., 2010). However although the techniques employed here (both DGGE and clone libraries) do not bias against vibrios, which we routinely detect in other coral diseases (for example in YBD, A. Croquer, A. Elliot, C. Bastidas and M.J. Sweet, unpublished), here only one strain of vibrio closely related to *V. harveyi* (JN406288) was identified. This ribotype increased in relative 16S rRNA gene abundance in diseased samples but was also present in non-diseased, apparently healthy coral. None of the strains of vibrio related to *V. coralliilyticus* implicated in WS by Sussman and colleagues (2008) was detected, although several other γ-proteobacteria were common in both the clone libraries and the DGGEs in this study. At present, we cannot rule out either ciliates and/or bacteria as causal agents, but the strong similarities in both bacterial and ciliate communities associated with PWS and BrB and observations of similar pathogenesis by the Morph1 ciliate (JN626268) in both diseases, strongly suggests that they are the same disease and we recommend that they be synonymized in future.

In this study we have applied a culture-independent approach to establish the microbial diversity (bacteria and ciliates) associated with two common coral diseases. In order to allow sufficient sample replication for statistical analysis, within reasonable costs, we used a DGGE screening approach which highlighted a number of dominant bacterial and ciliate ribotypes consistently associated with disease lesions from different host species and environments. The ciliate-specific DGGE screening successfully identified ribotypes matching all of the morphotypes microscopically observed and categorized using morphological characters (Lee *et al*., 2000), suggesting that at least in this relatively low-diversity community, the DGGE approach is accurate. For the higher diversity of bacterial communities, greater coverage was achieved through generation of six clone libraries from a variety of samples. More complete coverage could have been achieved via high-throughput sequencing (Mouchka *et al*., 2010). However, for potential pathogen identification, the focus was on the dominant ribotypes consistently present across many diverse samples rather than rarer amplicons and at present clone libraries can provide longer sequence reads providing greater phylogenetic resolution. In addition, all dominant ribotypes identified in the DGGE screening were also detected consistently in clone libraries, with the two techniques using independent primer sets targeting different 16S rRNA gene subregions, which suggest that the effects of potential primer bias were minimized.

Although this study was aimed at identifying the diversity of bacteria and ciliates associated with these coral diseases, and disease causation cannot be tested using a purely culture-independent approach, the observations lead us to propose two alternative hypotheses for causation of BrB/PWS. (i) Bacteria are the primary causal agents, invading healthy tissue and leading to an impaired physiological condition that allows ciliate communities to invade and proliferate at the lesion boundary, consuming health-compromised coral tissues. In this instance, two candidate bacterial pathogens were seen to increase in the apparently healthy tissues in advance of the disease lesion: *Arcobacter* sp. (JF831360) and *Aeromonas* sp. (JN406293). Due to the lack of consistency among samples and lower levels of upregulation of other bacterial pathogens (e.g. *V. harveyi*, *Glycomyces* sp., *Pseudoalteromonas* sp., *Shewanella* sp. and *Marinobacter* sp.), we propose that these are more likely secondary invaders of dead and decaying tissues following pathogenesis. Alternatively, (ii) ciliates are the causal agents and the bacterial agents identified are either pathogens that infect the host after it becomes physiologically stressed as a result of ciliate pathogenesis, or opportunistic heterotrophs invading dead and decomposing tissues. This latter hypothesis is supported by the observation that ciliates are completely absent from healthy coral and by previous studies (Ainsworth *et al*., 2007; Work and Aeby, 2011) which have not detected significant bacterial populations in the apparently healthy tissues at the advancing lesion edge. Work and Aeby (2011) particularly point out a lack of evidence for ‘bacterial-induced necrosis’ in the WS pathology. Under either of these hypotheses, while bacteria may represent a systemic infection, the ciliate communities reported in this study appear to be responsible for the characteristic visible signs of PWS/BrB, namely a rapidly advancing white band of denuded skeleton.

## Experimental procedures

In order to ensure that only active diseases were sampled, apparently diseased corals were tagged and photo-monitored over 4 days and only those showing lesion progression were subsequently sampled and analysed. All BrB lesions were found to progress whereas 83% of WS cases were progressive, which are referred to here as PWS.

To test for differences in bacterial and ciliate molecular diversity between healthy and PWS samples, we analysed coral fragments (∼ 2 cm length) from *n* = 10 non-diseased (ND) samples, *n* = 10 PWS samples (Fig. 1A) at the disease lesion interface and *n* = 10 apparently healthy (AH) tissues adjacent to the disease lesion for a single species, *A. muricata*, at Heron Island, GBR. In addition to this, we sampled from different host coral species, disease signs and locations in order to identify the bacterial and ciliate agents consistently and uniquely associated with disease lesions. Three specific contrasts were made, these were between: (i) PWS (*n* = 10, as above) and BrB lesions (*n* = 12) on *A. muricata* from Heron Island, GBR (Fig. 1B), (ii) PWS (*n* = 10, as above) on *A. muricata* and PWS on *A. aspera* (*n* = 4) from Heron Island, GBR, and (iii) PWS (*n* = 10, as above) on *A. muricata* from Heron Island, GBR and PWS (*n* = 5) on *A. muricata* from Solomon Islands. Samples were placed immediately into 50 ml falcon tubes and the water replaced with 100% EtOH and stored at −20°C until extraction and analysis.

### Bacterial 16S rRNA gene diversity

#### PCR amplification and DGGE of whole coral samples

All coral fragments (as above) were crushed using sterile, autoclaved pestle and mortar and DNA extracted using the Qiagen DNeasy Blood and Tissue Kit; spin column protocol (Sweet *et al*., 2010). For DGGE analysis a portion of the bacterial 16S rRNA gene was amplified using universal eubacterial primers: (357F) (5′-CCTACGGGAGGCAGCAG-3′) and (518R) (5′-ATTACCGCGGCTGCTGG-3′). The GC-rich sequence 5′-CGC CCG CCG CGC GCG GCG GGC GGG GCG GGG GCA GCA CGG GGG G-3′ was incorporated into the forward primer 357 at its 5′ end to prevent complete disassociation of the DNA fragments during DGGE. All reactions were performed using a Hybaid PCR Express thermal cycler. PCR reaction mixtures and programme were as described by Sweet and colleagues (2010). PCR products were verified by agarose gel electrophoresis [1.6% (w/v) agarose] with ethidium bromide staining and visualized using a UV transilluminator. DGGE was performed using the D-code universal mutation detection system (Bio-Rad). PCR products were resolved on 10% (w/v) polyacrylamide gels that contained a 30–60% formamide (denaturant) gradient for 13 h at 60°C and a constant voltage of 50 V. Gels were stained as described by Sweet and colleagues (2010). To identify the dominant DGGE bands across samples, representative bands (*n* = 21) were excised and sequenced to account for known DGGE artefacts such as heteroduplexes (Muyzer, 1999). Excised bands were left overnight in Sigma molecular grade water, vacuum centrifuged, re-amplified with primers 357F and 518R, labelled using Big Dye (Applied Biosystems) transformation sequence kit and sent to Genevision (Newcastle University UK) for sequencing. Bacterial operational taxonomic units (OTUs) were defined from DGGE band-matching analysis using Bionumerics 3.5 (Applied Maths BVBA) as described by Sweet and colleagues (2010).

#### Clone libraries and ARDRA screening

Almost-complete 16S rRNA gene fragments were amplified from the DNA extracted using the ‘universal’ eubacterial 16S rRNA gene primers 27F (5′-AGA GTT TGA TCG TGG CTC AG-3′) and 1542R (5′-AAG GAG GTG ATC CAG CCG CA-3′) (Cooney *et al*., 2002; Galkiewicz and Kellogg, 2008). Ten PCR cycles were performed at 94°C for 1 min, 55°C for 1 min and 72°C for 3 min then a further 25 cycles at 94°C for 1 min, 53°C for 1 min and 72°C for 3 min with a final extension at 72°C for 10 min. The amplified products were purified using the Qiagen PCR purification kit, inserted into the pGEM-T vector system (Promega) and transformed into *Escherichia coli* JM109 cells. A total of 392 clones containing the 16S rRNA gene inserts were randomly selected from each sample (non-diseased *A. muricata*, apparently healthy *A. muricata*, PWS *A. muricata*[GBR], PWS *A. aspera*[GBR], PWS *A. muricata*[Solomons] and BrB *A. muricata*), and boiled lysates were prepared from each by mixing a picked clone in 30 µl of TE and boiled for 3 min followed by freezing. Each lysate (1 µl) was amplified using the primers pUCF (5′-CTA AAA CGA CGG CCA GT-3′) and pUCR (5′-CAG GAA ACA GCT ATG AC-3′). Twenty-five PCR cycles were performed at 94°C for 1 min, 55°C for 1 min and 72°C for 1 min with a final extension at 72°C for 10 min. The products were then digested with the restriction enzymes HaeIII and RsaI (Promega) [4 µg of PCR product, 2 µl of restriction buffer, 0.2 µl of Bovine serum albumin (BSA), 0.07 µl of HaeIII, 0.1 µl of RsaI and made up to 20 µl with sigma water for 2 h at 37°C then 10 min at 67°C]. Restriction fragments were resolved by 3% agarose gel electrophoresis, visualized using a UV transilluminator and grouped based on restriction patterns. Representatives from each group were sequenced. Closest match of retrieved sequences was determined by RDP II similarity matching (Cole *et al*., 2009) Out of 162 clones sequenced, 52 unique sequences were retrieved from the six clone libraries, all sequences in this study have been deposited in GenBank and their unique accession numbers reported in the text.

### Ciliate 18S rRNA gene diversity

#### PCR amplification of single cell isolates

Ninety-three single cell isolates of the 11 different ciliate morphs visually identified on *A. muricata* exhibiting signs of both PWS and BrB at Heron Island were taken from mixed samples under binocular microscopy using a micropipette and preserved in 100% Analar ethanol. DNA was extracted from the ethanol-fixed single isolates using a modified Chelex extraction (Walsh *et al*., 1991). All samples were vacuum-centrifuged for 10 min and washed twice in Sigma water with a 2 min centrifuge step (20 000 *g*) in between. Following the final wash, 50 µl of 5% Chelex 100 (sigma) solution and 15 µl of proteinase K (20 mg ml^−1^) were added to the cell isolate. The samples were subsequently left in a water bath overnight at 54°C. After incubation, they were vortexed for 20 s, boiled at 100°C for 10 min, vortexed for a further 20 s and centrifuged at 16 000 *g* for 3 min. Thirty microlitres of supernatant was taken off and put in a fresh microcentrifuge tube. This was then stored at −20°C until further use. Twenty microlitres of PCR reactions were routinely used [final PCR buffer contained: 1 mM MgCl_2_, and 1 U *Taq* DNA polymerase (QBiogene); 100 µM dNTPs; 0.2 µM of each of the forwardand reverse primers; and 0.4% BSA, with 20 ng of template DNA extracted as above] in a Hybaid PCR-Express thermal cycler. The universal 18S rRNA gene eukaryotic primers 4617f (5′-TCCTGCCAGTAGTCATATGC-3′) and 4618r (5′-GATCCTTCTGCAGGTTCACC TAC-3′) (T. Tengs, pers. comm.) were used following the PCR protocol of Oldach and colleagues (2000). The nested PCR reaction was carried out using 1 µl of a 1:100 dilution of the first round PCR product with the ciliate-specific primers 384f-cil (5′-YTBGATGGTAGTGTATTGGA-3′) and 1147r-cil (5′-GACGGTATCTRATCGTC TTT-3′), amplification conditions followed that of Dopheide and colleagues (2008). All sequences were ethanol-purified from PCR products and sequenced as above.

#### PCR amplification and DGGE of whole coral samples

From crushed and extracted samples, ciliate 18S rRNA genes were amplified with an un-nested PCR approach (Jousset *et al*., 2010). Three 10 µl replicates of each sample were run using 8 ng of DNA product (PCR mixture as above) with the forward primer CilF (5′-TGGTAGTGTATTGGACWACCA-3′) with a 36 bp GC clamp (Muyzer and Smalla, 1998) attached to the 5′ end and CilDGGE-r (5′-TGAAAACATCCTTGGCAACTG-3′). Initial denaturation was at 94°C for 5 min, followed by 26 cycles of 94°C for 1 min, 52°C for 1 min and 72°C for min and a final elongation step of 10 min at 72°C to reduce double bands in the DGGE patterns (Janse *et al*., 2004). The three PCR products of each sample were combined and DGGE carried out using a D-code system (Bio-Rad) with 0.75 mm thick 6% polyacrylamide gels in 1× TAE buffer. Electrophoresis was carried out for 16 h at 60°C and 50 V in a linear 32–42% denaturant (formamide) gradient. Gels were stained with SYBR Gold as above.

### Microscopic observation and characterization of the dominant ciliates

Additional coral fragments showing signs of PWS (*n* = 5) and BrB (*n* = 5) on *A. muricata* and PWS on *A. aspera* (*n* = 5) were collected from Heron Island reef, GBR and transferred without handling to an observation tank for microscopic and behavioural observations of associated ciliate species using an Olympus SZX7 binocular microscope and Olympus LG-PS2 fibre-optic light source. Still images and time-lapse videos were captured using a QImaging Micropublisher 3.3 camera and Q-Capture v6 imaging software. Higher-magnification images were obtained using an Olympus BX51 compound microscope and images captured as above. The images were compared with morphological descriptions in previous studies (Carey, 1992; Lee *et al*., 2000; Song, 2000; Croquer *et al*., 2006b; Page and Willis, 2008; Shimano *et al*., 2008), alongside the use of a dichotomous key in the ‘Illustrated Guide to the Protozoa’ (Lee *et al*., 2000). Morphological characteristics, such as cortical and ultrastructural features, provided a means of distinguishing ciliate morphotypes. Features such as kinetosomal make-up and oral infraciliary structures such as the AZM (Adoral Zone of Membranelles) are highly conserved features and together with organelle distribution, size, shape and colour are routinely used for distinguishing genera (Lee *et al*., 2000).

### Statistical analysis

A one-way ANOSIM based on Bray-Curtis similarities of band intensity patterns was performed to test for differences between DGGE profiles of the bacterial 16S rRNA and ciliate 18S rRNA gene assemblages associated with different coral species, locations and health states using PRIMER v6 (Clarke and Warwick, 2001). Pairwise comparisons within ANOSIM were used to contrast between specific sample types (Anderson, 2001).

## Supporting information

**Fig. S1.** Regions of the alignment of partial subunit 18S rRNA gene sequences from the dominant ciliates [Morph1(JN626268) and Morph2 (JN626269)] seen in PWS and BrB, along with closest relatives available in GenBank. Reference sequence is given at the top and colour coded for individual bases. Insertions were compensated by introducing alignment gaps (−). Matched sitesare represented in dots (.). Distinct sequence signatures of each ciliate aligned are indicated in colour according to base: T = green; A = red; G = yellow; and C = blue.

**Video S1.** Time-lapse microvideography of lesion progression in White Syndrome. A diverse ciliate community can been seen massing at the edge and underneath the tissues at the lesion edge. Polychaete worms can also be seen predating on the ciliates. Individual ciliate cells of the smaller morphotype, Morph1 (Table 2) cannot be seen at this magnification and appear as yellowish masses. Video sequence is 300 frames at a sampling rate of 3 min per frame; refer to Fig 3 for scale.

**Video S2.** Time-lapse microvideography of lesion progression in Brown Band Disease. A diverse ciliate community can been seen massing at the edge of the disease lesion. Individual ciliate cells of the smaller morphotype; Morph1 (Table 2) cannot be seen at this magnification and appear as a yellowish diffuse mass. Individual cells of the larger morphotype, Morph2 (Table 2), which gives rise to the characteristic brown band visible to the naked eye, can be seen in abundance. Video sequence is 300 frames at a sampling rate of 3 min per frame; refer to Fig 3 for scale.
