# Supplementary material for: Ciliate and bacterial communities associated with White Syndrome and Brown Band Disease in reef-building corals
Source: Environ Microbiol. 2012 Aug;14(8):2184–99. doi: 10.1111/j.1462-2920.2012.02746.x (PMC3465780; doi:10.1111/j.1462-2920.2012.02746.x)
Supplement: Supplementary file 1 [file emi0014-2184-SD1.pptx]

## Slide 1
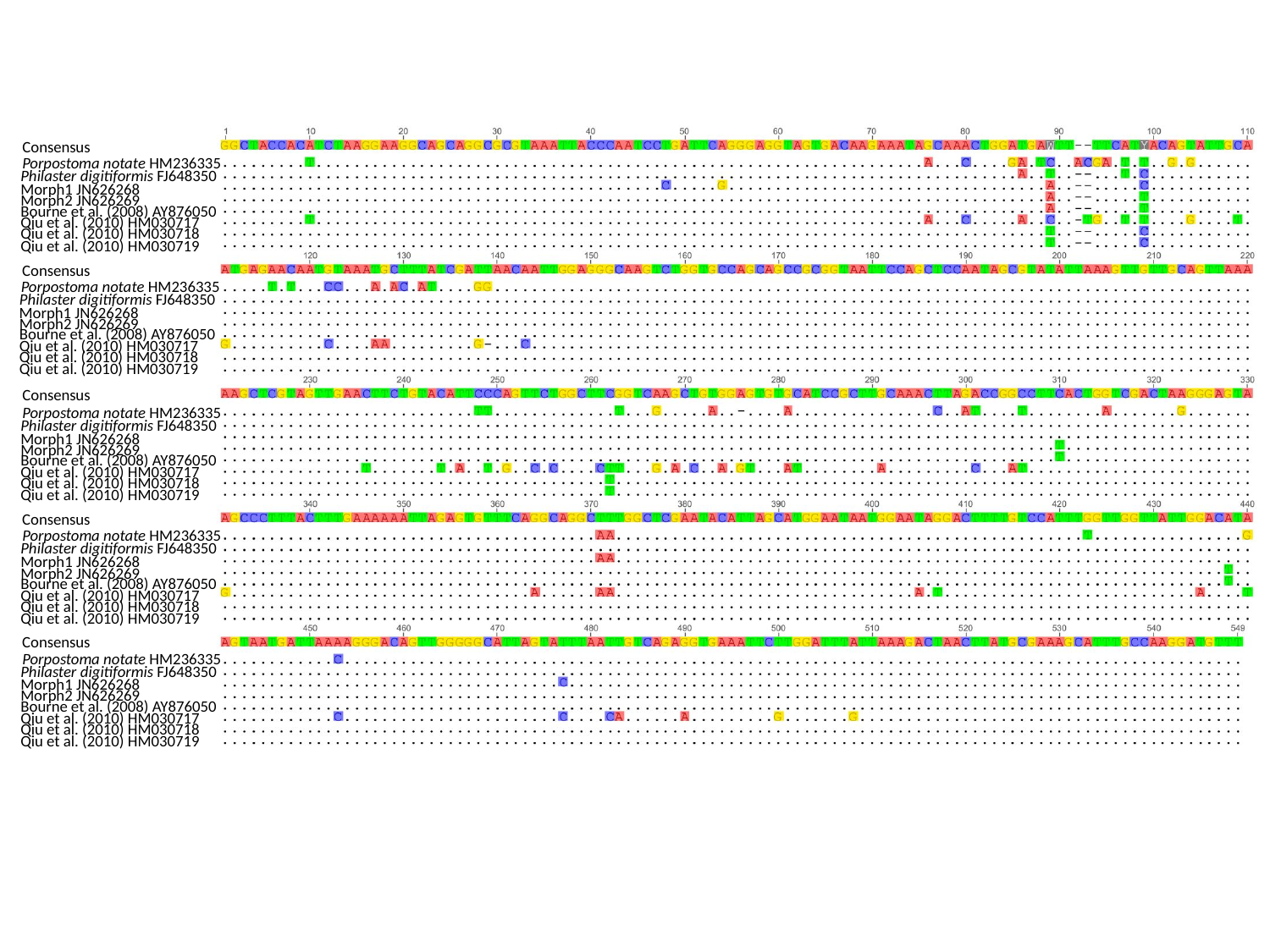

Consensus
Porpostoma notate HM236335
Philaster digitiformis FJ648350
Morph1 JN626268
Morph2 JN626269
Bourne et al. (2008) AY876050
Qiu et al. (2010) HM030717
Qiu et al. (2010) HM030718
Qiu et al. (2010) HM030719
Consensus
Porpostoma notate HM236335
Philaster digitiformis FJ648350
Morph1 JN626268
Morph2 JN626269
Bourne et al. (2008) AY876050
Qiu et al. (2010) HM030717
Qiu et al. (2010) HM030718
Qiu et al. (2010) HM030719
Consensus
Porpostoma notate HM236335
Philaster digitiformis FJ648350
Morph1 JN626268
Morph2 JN626269
Bourne et al. (2008) AY876050
Qiu et al. (2010) HM030717
Qiu et al. (2010) HM030718
Qiu et al. (2010) HM030719
Consensus
Porpostoma notate HM236335
Philaster digitiformis FJ648350
Morph1 JN626268
Morph2 JN626269
Bourne et al. (2008) AY876050
Qiu et al. (2010) HM030717
Qiu et al. (2010) HM030718
Qiu et al. (2010) HM030719
Consensus
Porpostoma notate HM236335
Philaster digitiformis FJ648350
Morph1 JN626268
Morph2 JN626269
Bourne et al. (2008) AY876050
Qiu et al. (2010) HM030717
Qiu et al. (2010) HM030718
Qiu et al. (2010) HM030719
